# MiR-17-5p and miR-20a promote chicken cell proliferation at least in part by upregulation of c-Myc via MAP3K2 targeting

**DOI:** 10.1038/s41598-017-15626-9

**Published:** 2017-11-20

**Authors:** Xiaofei Zhang, He Song, Shupei Qiao, Jing Liu, Tianyu Xing, Xiaohong Yan, Hui Li, Ning Wang

**Affiliations:** 1 Key Laboratory of Chicken Genetics and Breeding, Ministry of Agriculture, Harbin, 150030 Heilongjiang China; 2 Key Laboratory of Animal Genetics, Breeding and Reproduction, Education Department of Heilongjiang Province, Harbin, 150030 Heilongjiang China; 3 Key Laboratory of Animal Cells and Genetic Engineering of Heilongjiang Province, Harbin, 150030 Heilongjiang China

## Abstract

The miR-17-92 cluster has been well studied in mammals but less extensively studied in birds. Here, we demonstrated that miR-17-92 cluster overexpression promoted the proliferation of DF1 cells and immortalized chicken preadipocytes (ICPA-1), and miR-17-5p and miR-20a, members of the miR-17-92 cluster, targeted MAP3K2. Further analysis showed that MAP3K2 overexpression reduced the proliferation of DF1 and ICPA-1 cells and attenuated the promotive effect of the miR-17-92 cluster on cell proliferation. Downstream gene expression analysis of the MAPK signalling pathway showed that MAP3K2 overexpression decreased c-Myc expression; in contrast, MAP3K2 knockdown using RNA interference and miR-17-92 cluster overexpression increased c-Myc expression. Furthermore, c-Myc overexpression promoted miR-17-92 cluster expression and DF1 cell proliferation. Taken together, these data indicated that miR-17-92 promotes chicken cell proliferation at least in part by the upregulation of c-Myc via targeting MAP3K2, and the miR-17-92 cluster, c-Myc and E2F1 form a complex regulatory network in chicken cell proliferation.

## Introduction

MicroRNAs (miRNAs) are a class of small (19–25 nucleotides in length) endogenous non-coding RNAs that function as important post-transcriptional gene regulators. MiRNAs negatively regulate gene expression through translational repression or mRNA degradation by base-pairing to the 3′-untranslated region of target mRNAs and play vital roles in diverse physiological and pathological processes, such as cell proliferation, differentiation, apoptosis, development and cancer^1,2^. MiRNAs are non-randomly distributed over the genome, and many miRNAs are clustered on chromosomes^3^. The human miR-17-92 cluster, a well-characterized miRNA cluster, is located in the third intron of the miR-17-92 host gene (MIR17HG)^4^. The miR-17-92 cluster can generate at least six mature miRNAs (miR-17, miR-18a, miR-19a, miR-20a, miR-19b-1 and miR-92a-1) from the same primary transcript^5^.

The miR-17-92 cluster is widely expressed in embryo and adult tissues and plays important roles in various physiological and pathological processes. Knockout mouse studies have demonstrated that the miR-17-92 cluster is essential for lung, cardiogenesis, and skeletal development^6,7^. Transgenic mouse studies have revealed that miR-17-92 cluster overexpression in lung epithelium enhanced proliferation and inhibited differentiation^8^. In addition, miR-17-92 cluster overexpression increased triglyceride accumulation and accelerated 3T3-L1 preadipocyte differentiation^9^. The miR-17-92 cluster is highly expressed in multiple tumour types and promotes tumour growth in human and mouse cell models^10^. The miR-17-92 cluster is the first characterized oncomiR, termed oncomir-1^11^. This cluster inhibits the expression of tumour suppressor genes (p21, PTEN, Bim and RB1)^12–15^, cell cycle regulator genes (E2F family)^16,17^, and anti-angiogenesis-related factors CTGF and TSP-1 and promotes tumour cell proliferation^18^. However, several studies have demonstrated that the miR-17-92 cluster also functions as a tumour suppressor. For example, the miR-17-92 cluster inhibits the progression of colorectal cancer by targeting angiogenesis^19^.

Accumulating evidence has revealed that the miR-17-92 cluster functions via targeting distinct signalling pathways, such as MAPK, TGFβ, Wnt/β-catenin, and Hedgehog signalling pathways, depending on the tissue and cell types^20–22^. Mitogen-activated protein kinase kinase kinase 2 (MAP3K2, also known as MEKK2) is a member of the MEK kinase (MEKK) group of MAP3Ks^23^. MAP3K2 is an upstream MAPK kinase kinase of MAPK signalling pathway, which plays crucial roles in cell proliferation, differentiation, and cell migration^24^. MAP3K2 can activate several downstream kinases of the MAPK signalling pathway, including ERK1/2, JNK, p38, and ERK5^25,26^. RNA interference analysis showed that MAP3K2 promoted lung cancer cell proliferation, migration and invasion and inhibited cell apoptosis *in vitro* ^27^. However, in glioblastoma cells, MAP3K2 acts as a tumour suppressor and promotes apoptosis^28–30^.

Multiple roles and molecular mechanisms of the miR-17-92 cluster have been revealed in mammals; however, there is little information concerning the miR-17-92 cluster in birds. A recent study showed that gga-miR-19b-3p, a member of the miR-17-92 cluster, was significantly differentially expressed in preadipocytes between chickens with high and low abdominal adipose weight, and further studies showed that gga-miR-19b-3p targeted ACSL1 (acyl-CoA synthetase long-chain family member 1), and overexpression of gga-miR-19b-3p promoted chicken preadipocyte proliferation and differentiation^31^. Previous Solexa sequencing showed that the miR-17-92 cluster was expressed in chicken preadipocytes of Arbor Acres broiler^32^ and Northeast Agricultural University broiler lines divergently selected for abdominal fat content (NEAUHL). In the present study, we investigated the effect and potential mechanism of the miR-17-92 cluster on chicken cell proliferation, and the results demonstrated that the miR-17-92 cluster promotes chicken cell proliferation, at least in part, by increasing the expression of c-Myc via targeting MAP3K2.

## Results

### MiR-17-92 cluster expression in chicken cells

**Figure 1:**
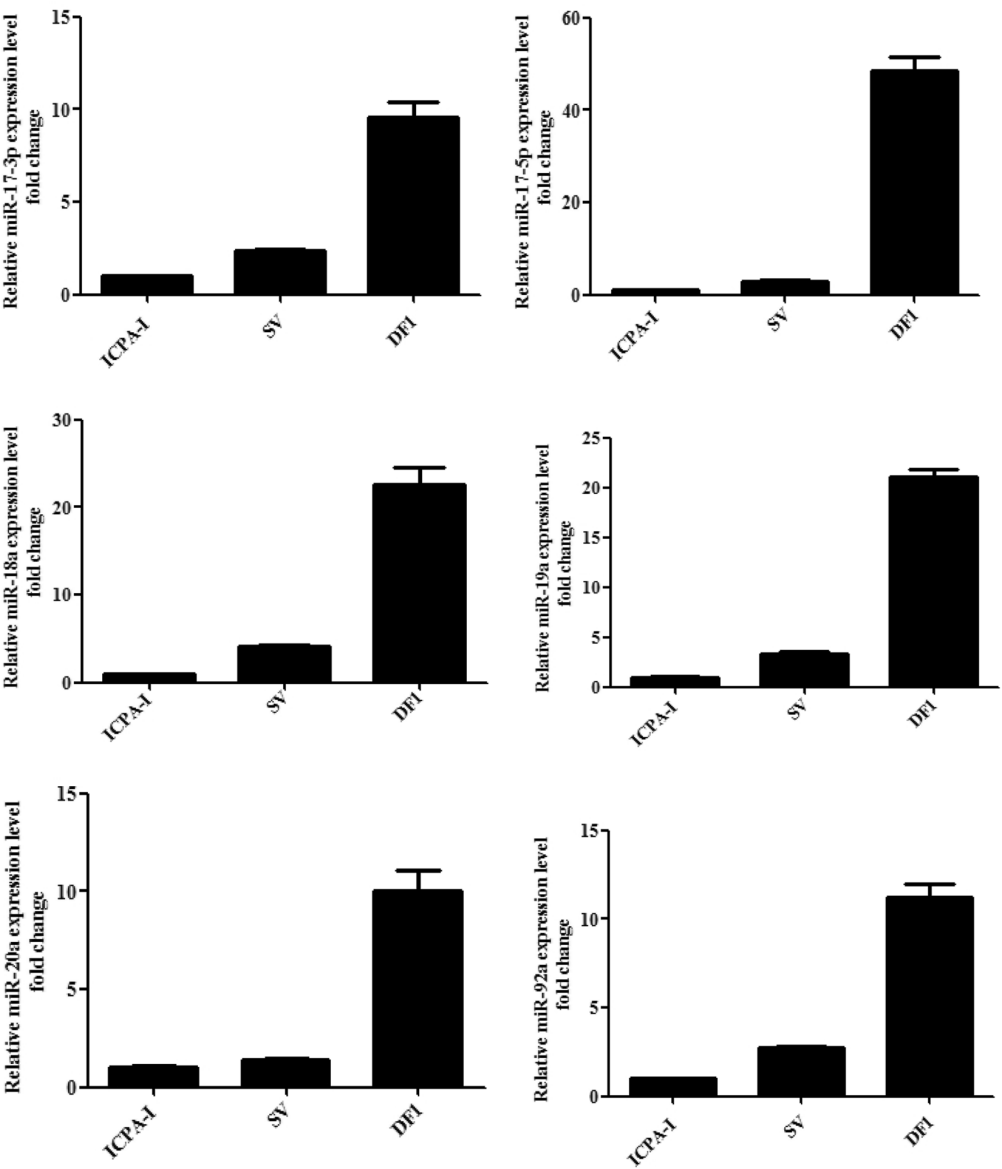
The relative expression of individual members of the miR-17-92 cluster in three chicken cell lines. The relative miRNA expression was analysed using stem-loop qRT-PCR, and miRNA expression was normalized to the U6 snRNA expression level. Fold change is relative to ICPA-1 cells. All data are representative of three independent experiments and shown as the mean ± SEM. Abbreviation: ICPA-1, immortalized chicken preadipocyte cell line 1; SV, stromal-vascular cell fraction (chicken preadipocytes).

The results of a previous Solexa sequencing study showed that the miR-17-92 cluster was expressed in chicken preadipocytes of Arbor Acres broiler and the divergently selected lean and fat broilers of Northeast Agricultural University (NEAUHLF)^32,33^. To understand the role of the miR-17-92 cluster in chicken cells, we detected the expression of individual members of miR-17-92 cluster in DF1 cells, immortalized chicken preadipocytes (ICPA-1) and freshly isolated chicken preadipocytes (stromal-vascular cell fraction, SV) from the abdominal adipose tissue of the broilers of NEAUHLF using stem-loop qRT-PCR. The results showed that, consistent with the Solexa sequencing results, miR-17-92 cluster members were expressed in chicken preadipocytes (Fig. 1). Among these three different cells, the members of miR-17-92 cluster (miR-17-3p, miR-17-5p, miR-18a, miR-19a, miR-20a and miR-92a) were more highly expressed in DF1 cells than in SV and ICPA-1 cells (*p* < 0.01) (Fig. 1). Considering that DF1 cells, a continuous cell line of chicken embryo fibroblasts, are highly proliferative compared with SV and ICPA-1 cells, we presumed that the miR-17-92 cluster might promote chicken cell proliferation.

### MiR-17-92 cluster promotes chicken cell proliferation

**Figure 2:**
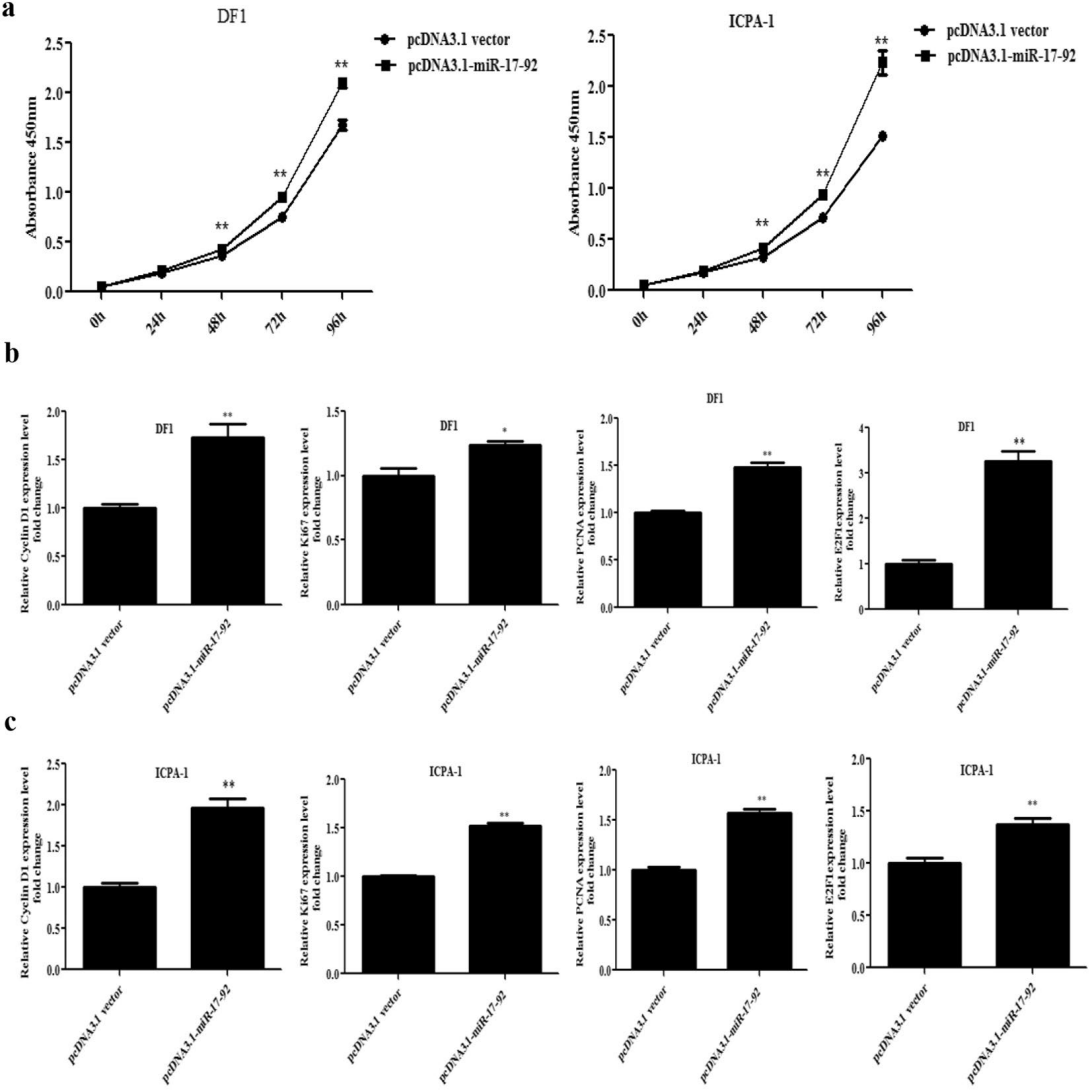
Overexpression of miR-17-92 cluster promotes proliferation of DF1 and ICPA-1 cells. (**a**) Effect of overexpression of miR-17-92 cluster on chicken cell proliferation. DF1 and ICPA-1 cells were transfected with either pcDNA3.1-miR-17-92 or the empty vector pcDNA3.1, and cell proliferation was assessed at the designated time points using the CCK-8 kit. (**b** and **c**) Effect of overexpression of miR-17-92 cluster on expression of proliferation marker genes in DF1 cells (**b**) and ICPA-1 cells (**c**). Cells were transfected with either pcDNA3.1-miR-17-92 or pcDNA3.1 vector, total RNA was isolated, and the gene expression of Cyclin D1, Ki67, PCNA and E2F1 were assessed at 48 h post-transfection using qRT-PCR. Gene expression was normalized to NONO mRNA level. Fold change is relative to pcDNA3.1 vector at 48 h after transfection. All data are representative of three independent experiments and shown as the mean ± SEM. *p < 0.05; **p < 0.01; determined by two-tailed Student’s t-test.

Mammalian studies have shown that miR-17-92 cluster promotes cell proliferation in a variety of cell types^6–10^. To examine whether miR-17-92 cluster promotes chicken cell proliferation, DF1 and ICPA-1 cells were transfected with either a vector expressing miRNA-17-92 cluster (pcDNA3.1-miR-17-92 cluster) or a control vector (pcDNA3.1 vector), and cell proliferation was assayed using the CCK-8 assay. The results showed that in both DF1 and ICPA-1 cells, the absorbance values (OD) of the cells transfected with the pcDNA3.1-miR-17-92 cluster were significantly higher than those of cells transfected with the pcDNA3.1 vector at 48, 72, and 96 h after transfection (*p* < 0.01) (Fig. 2a). These data suggest that miR-17-92 cluster promotes the proliferation of DF1 and ICPA-1 cells. Furthermore, we detected the effect of miR-17-92 cluster overexpression on the expression of proliferation markers (Cyclin D1, Ki67, PCNA and E2F1) using qRT-PCR. The results showed, at 48 h post-transfection, compared with the control pcDNA3.1 vector, overexpression of miR-17-92 cluster increased the expression of Cyclin D1, Ki67, PCNA and E2F1 nearly 73.6%, 23.6%, 48.9%, and 228%, respectively, in DF1 cells (Fig. 2b and Supplementary Fig. S1a), and 96.2%, 52.6%, 57.0%, and 37.2%, respectively, in ICPA-1 cells (Fig. 2c and Supplementary Fig. S1b), consistent with the above CCK-8 results. Taken together, we concluded that miR-17-92 cluster promotes chicken cell proliferation.

### Bioinformatics analysis of the miR-17-92 cluster

**Figure 3:**
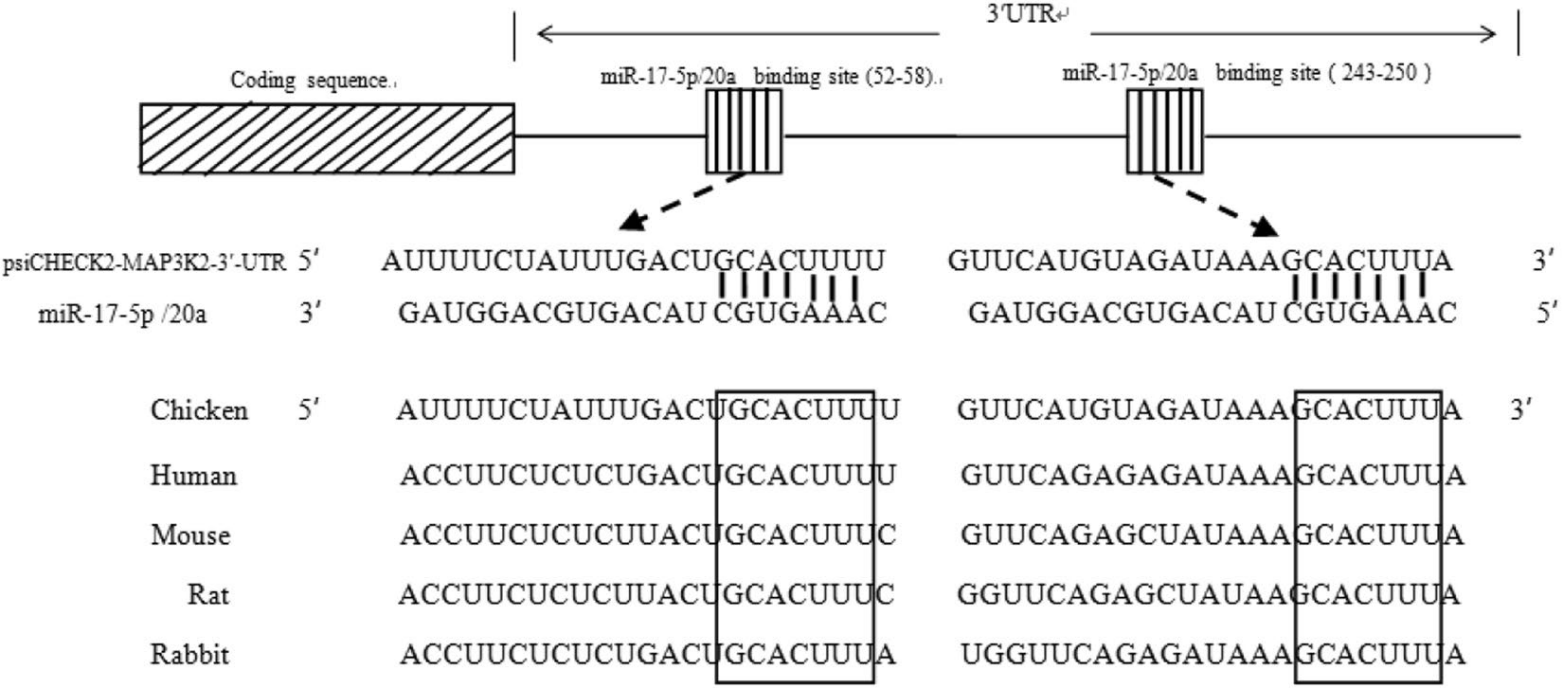
The predicted binding sites of miR-17-5p and miR-20a in the 3′ untranslated region (3′UTR) of chicken MAP3K2 mRNA. The figure shows that MAP3K2 has two putative binding sites for miR-17-5p/20a in its 3′UTR and the two putative binding sites are highly conserved in various animal species. The miRNA binding sites were predicted using TargetScan, miRanda and PicTar software. The boxed sequences indicate the putative binding sites for miR-17-5p and miR-20a in the 3′UTR of MAP3K2 mRNA.

To understand the molecular mechanism underlying the promotive effect of the miR-17-92 cluster on chicken cell proliferation, we performed miRNA target prediction using the three most commonly used online software programmes, miRanda (http://www.microrna.org/), TargetScan (http://www.targetscan.org/) and PicTar (http://pictar.mdc-berlin.de/). A number of genes were predicted to be targeted by the members of the miR-17-92 cluster, some of which have been experimentally verified in mammals, such as PTEN, Bim, and TGFBR2^12,14,15^. Herein, we focused on one of the predicted target genes, MAP3K2. MAP3K2 is a component of the MAPK signalling pathway, which plays vital roles in multiple cellular functions, including (but not limited to) cell survival, proliferation, differentiation and cell migration. Several previous studies have demonstrated that MAP3K2 is involved in the regulation of proliferation in a variety of cell types^27,34^. Bioinformatics analysis showed that chicken MAP3K2 mRNA contained two putative binding sites for miR-17-5p/20a at nucleotides 52-58 and 243-250 in its 3′UTR, and these two putative miRNA binding sites were conserved among chickens and other animal species (Fig. 3). Together, these data suggest that the miR-17-92 cluster may promote chicken cell proliferation in part via MAP3K2 targeting by its two members, miR-17-5p and miR-20a.

### MAP3K2 is targeted by miR-17-5p/20a

**Figure 4:**
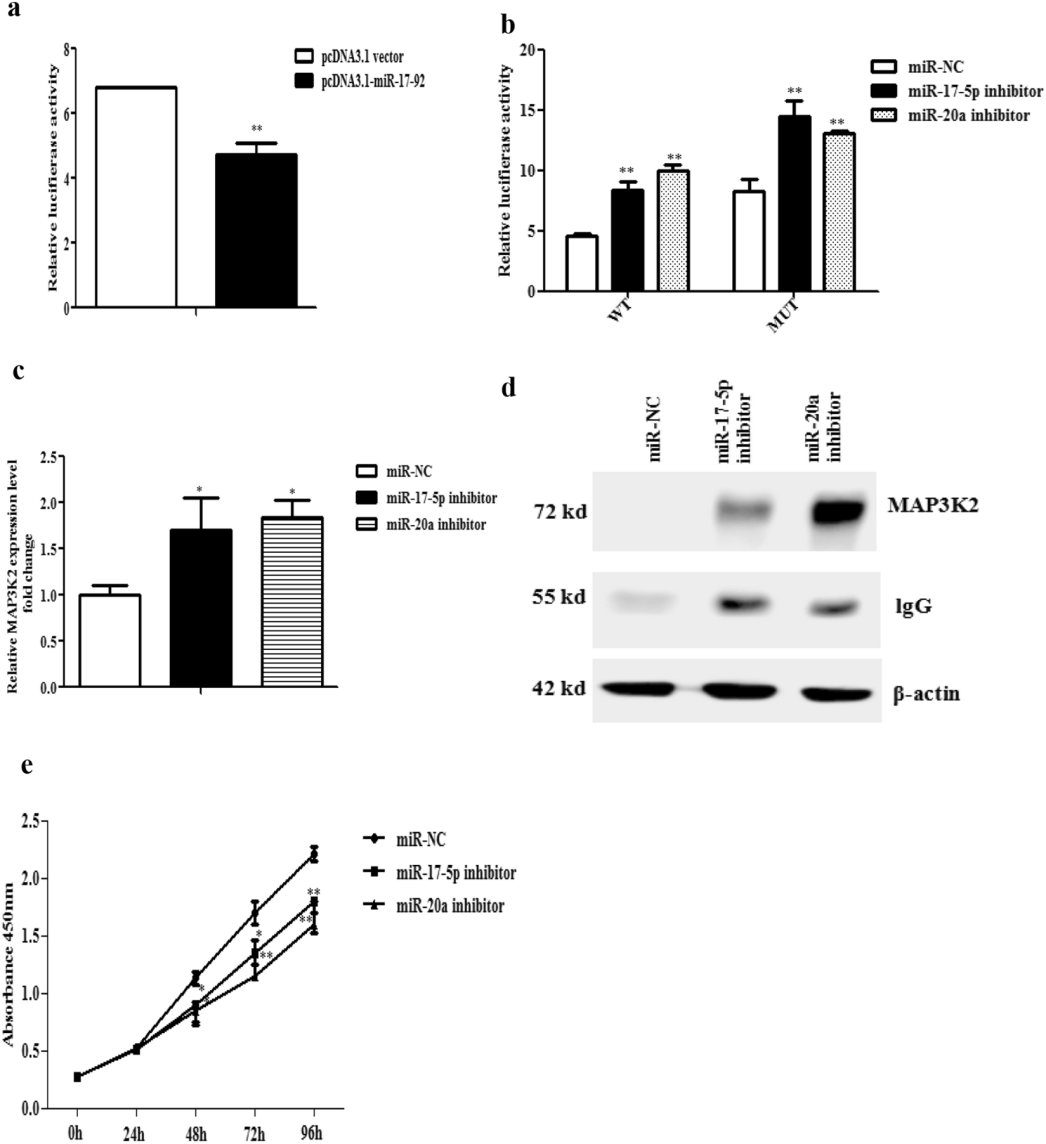
MAP3K2 is directly targeted using miR-17-5p/20a. (**a**) Effect of miR-17-92 cluster overexpression on the luciferase reporter activity of psi-CHECK2-MAP3K2-3′UTR-WT. DF1 cells were cotransfected with either pcDNA3.1-miR-17-92 or the pcDNA3.1 vector along with psi-CHECK2-MAP3K2-3′UTR-WT. The firefly and Renilla luciferase activities were assayed at 48 h after transfection. (**b**) Effect of miRNA inhibitors on the luciferase reporter activity of psi-CHECK2-MAP3K2-3′UTR-WT. DF1 cells were cotransfected with either psi-CHECK2-MAP3K2-3′UTR-WT or psi-CHECK2-MAP3K2-3′UTR-MUT with the indicated miRNA inhibitors. The firefly and Renilla luciferase activities were measured at 48 h after transfection. Relative luciferase activity was expressed as the ratio of renilla to firefly luciferase activity. (**c**) qRT-PCR analysis of MAP3K2 mRNA expression. DF1 cells were transiently transfected with the indicated miRNA inhibitors. MAP3K2 mRNA expression was assessed using qRT-PCR. MAP3K2 expression was normalized to NONO mRNA level. Fold change is relative to miR-NC (negative control) at 48 h after transfection. (**d**) IP-western blot analysis of MAP3K2 in DF1 cells. DF1 cells were transfected with the indicated miRNA inhibitors. At 48 h after transfection, the cells were harvested, and MAP3K2 protein expression was assessed using IP-western blot analysis. Matched inputs were assayed for β-actin using western blotting. (**e**) Effect of miR-17-5p and miR-20a inhibitors on the proliferation of DF1 cells. DF1 cells were transfected with the indicated miRNA inhibitors and miR-NC, and cell proliferation was assayed using the CCK-8 kit. All data are representative of three independent experiments and shown as the mean ± SEM. *p < 0.05; **p < 0.01; determined by two-tailed Student’s t-test.

To verify whether miR-17-5p and miR-20a target chicken MAP3K2, the 3′UTR fragment (598 bp) of chicken MAP3K2 mRNA, containing the two putative binding sites for miR-17-5p/20a, was RT-PCR amplified and cloned into the psi-CHECK2 vector to yield the MAP3K2 3′UTR reporter (psi-CHECK2-MAP3K2-3′UTR-WT). A reporter gene assay showed that overexpression of the miR-17-92 cluster highly significantly decreased the luciferase reporter activity of psi-CHECK2-MAP3K2-3′UTR-WT by 30.7% in DF1 cells (*p* < 0.01) compared with the control group (pcDNA3.1 vector) (Fig. 4a). In contrast, both miR-17-5p and miR-20a inhibitors highly significantly increased the luciferase activities of psi-CHECK2-MAP3K2-3′UTR-WT in DF1 cells (Fig. 4b). To further verify whether miR-17-5p/20a directly targets MAP3K2, four nucleotides within the two putative miRNA binding sites were mutated in the reporter psi-CHECK2-MAP3K2-3′UTR-WT to generate the MAP3K2 3′UTR mutant reporter (psi-CHECK2-MAP3K2-3′UTR-MUT). These two reporters (psi-CHECK2-MAP3K2-3′UTR-WT and psi-CHECK2-MAP3K2-3′UTR-MUT) were respectively transfected into DF1 cells, in which the miR-17-92 cluster was highly expressed (Fig. 1). The luciferase reporter results showed that the activity of psi-CHECK2-MAP3K2-3′UTR-MUT was significantly higher than that of psi-CHECK2-MAP3K2-3′UTR-WT, suggesting that these miR-17-5p and miR-20a directly target the 3′UTR of MAP3K2. Interestingly, we also observed that the miR-17-5p/20a inhibitor also significantly increased the luciferase activities of the mutant reporter (psi-CHECK2-MAP3K2-3′UTR-MUT) (Fig. 4b), likely reflecting the incomplete loss of interaction between these two miRNAs and their respective mutated binding sites.

To further understand the mode of action for miR-17-5p/20a in inhibiting MAP3K2 expression, miR-17-5p and miR-20a inhibitors were respectively transfected into DF1 cells, and MAP3K2 mRNA and protein expression were examined using qRT-PCR and IP-western blot analyses. As shown in Fig. 4c, d, compared with miR-NC (negative control), miR-17-5p and miR-20a inhibitors increased MAP3K2 mRNA by 70.1% and 84.5% (*p* < 0.05), respectively, at 48 h after transfection. Consistently, at 48 h after transfection, IP-western blot analysis showed that MAP3K2 protein expression was significantly elevated by the two miRNA inhibitors compared with miR-NC (Supplementary Fig. S2). These data suggest that miR-17-5p and miR-20a target MAP3K2 by inducing mRNA degradation.

### Inhibition of miR-17-5p and miR-20a reduces the proliferation of DF1 cells

To determine whether the miR-17-92 cluster promotes chicken cell proliferation through its two members, miR-17-5p and miR-20a, we tested the effect of miR-17-5p and miR-20a inhibitors on chicken cell proliferation. The miR-17-5p and miR-20a inhibitors were transfected into DF1 cells and cell proliferation was assayed using the CCK-8 kit. The results showed that both miR-17-5p and miR-20a inhibitors could inhibit the proliferation of DF1 cells compared with the negative control group (miR-NC) (Fig. 4e), in contrast with the effect of the miR-17-92 cluster on the proliferation of DF1 cells, suggesting that miR-17-5p and miR-20a mediate the promotive effect of the miR-17-92 cluster in the chicken cell proliferation.

### Overexpression of MAP3K2 decreases the proliferation of DF1 and ICPA-1 cells

**Figure 5:**
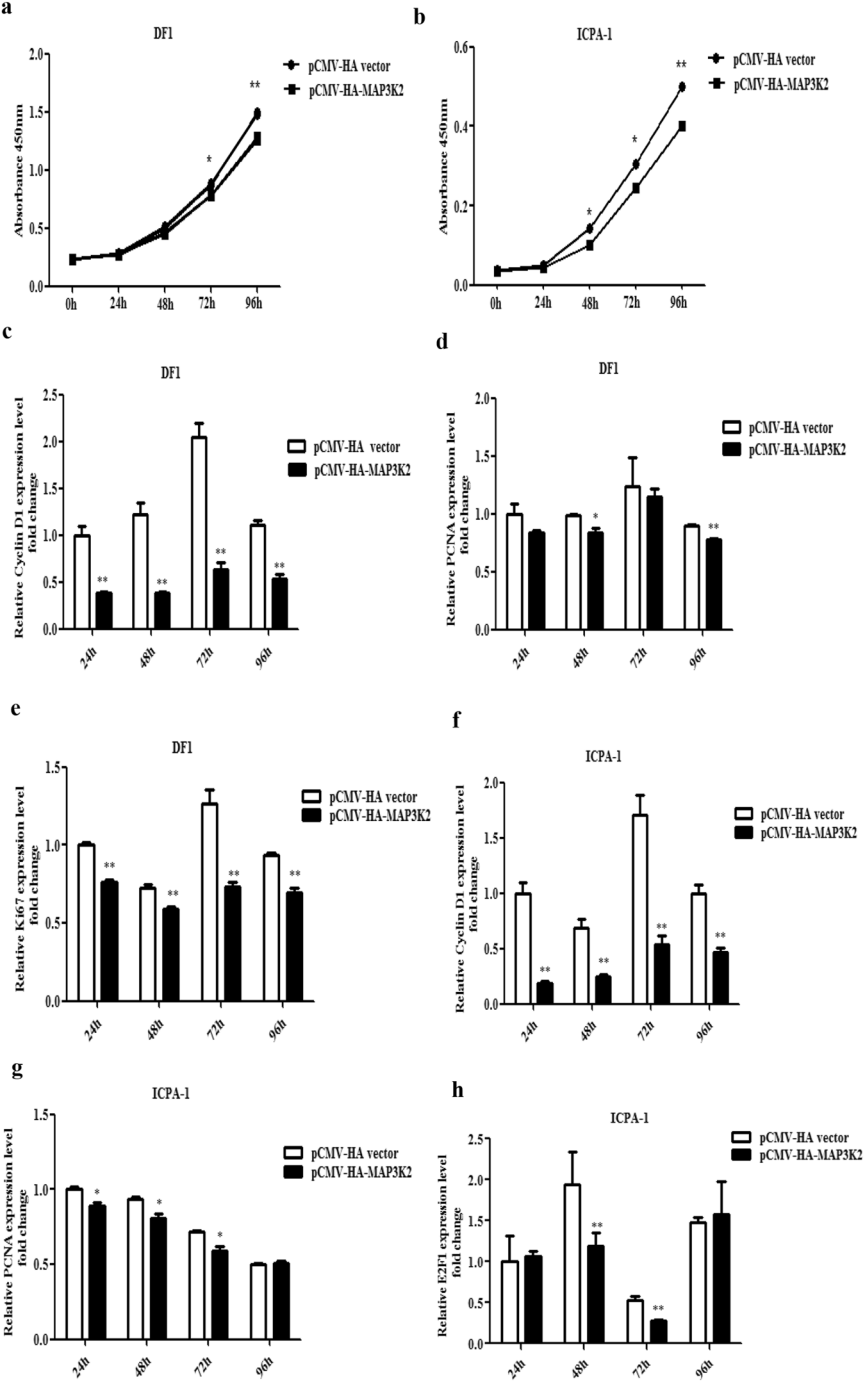
Overexpression of MAP3K2 inhibits chicken cell proliferation. (**a** and **b**) Effect of overexpression of MAP3K2 on proliferation of DF1 (**a**) and ICPA-1 cells (**b**). The cells were transfected with pCMV-HA-MAP3K2 or pCMV-HA vector, and cell proliferation was assayed at designated time points after transfection using the CCK-8 kit. (**c**–**h**) qRT-PCR analysis of Cyclin D1, PCNA, Ki67 or E2F1 in DF1 (**c**–**e**) and ICPA-1 cells (**f**–**h**). The cells were transfected with either pCMV-HA-MAP3K2 or pCMV-HA vector, and at designated time points after transfection, total RNA was isolated, and gene expression was assessed using qRT-PCR analysis. Gene expression was normalized to NONO mRNA level. Fold change is relative to pCMV-HA vector at 24 h after transfection. All data are representative of three independent experiments and shown as the mean ± SEM. *p < 0.05; **p < 0.01; determined by two-tailed Student’s t-test.

To determine the role of MAP3K2 in chicken cell proliferation, we tested the effect of MAP3K2 overexpression on the proliferation of DF1 and ICPA-1 cells. We cloned the full-length coding sequence of chicken MAP3K2 in a eukaryotic expression vector (pCMV-HA vector) to yield the MAP3K2 expression vector, pCMV-HA-MAP3K2. The CCK-8 assay showed that MAP3K2 overexpression markedly inhibited cell proliferation by 10.8% and 11.2%, respectively, at 72 and 96 h in DF1 cells (Fig. 5a) and by 29.0%, 19.8% and 20%, respectively, at 48, 72, and 96 h in ICPA-1 cells, compared with the pCMV-HA vector (Fig. 5b). In parallel, we also detected the expression of proliferation markers Cyclin D1, PCNA, Ki67 or E2F1 at 24, 48, 72 and 96 h after transfection. Consistent with the CCK-8 results, MAP3K2 overexpression significantly inhibited the expression of PCNA, Ki67 and CyclinD1 in DF1 cells at 24, 48, 72 and 96 h after transfection (*p* < 0.01 or *p* < 0.05) (Fig. 5c–e). Similarly, in ICPA-1 cells, the overexpression of MAP3K2 inhibited the expression of PCNA, Cyclin D1 and E2F1 (*p* < 0.01 or *p* < 0.05) (Fig. 5f–h). Taken together, these data suggest that MAP3K2 inhibits chicken cell proliferation.

### MAP3K2 overexpression does not induce cell apoptosis in DF1 and ICPA-1 cells

**Figure 6:**
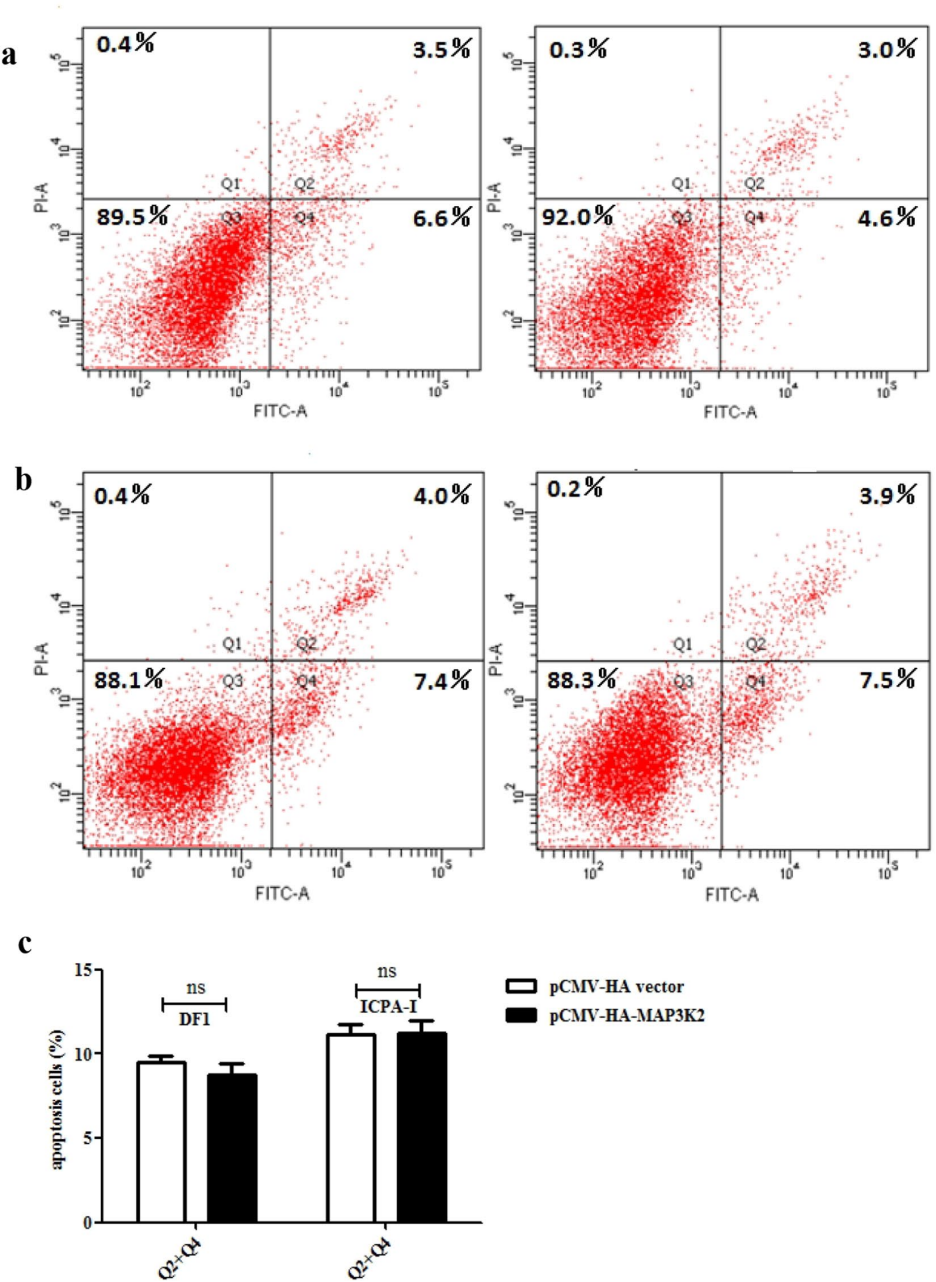
MAP3K2 overexpression does not induce cell apoptosis. (**a** and **b**) Effect of MAP3K2 overexpression on cell apoptosis. DF1 cells (**a**) and ICPA-1 cells (**b**) were transfected with either pCMV-HA-MAP3K2 or pCMV-HA vector, and at 48 h after transfection, cell apoptosis was assessed using the Annexin V-FITC/PI kit. The results are representative of three independent experiments. (**c**) The quantification of cell apoptosis in DF1 and ICPA-1 cells. All data are representative of three independent experiments and shown as the mean ± SEM, and ns indicates no significance; determined by two-tailed Student’s t-test.

The results of the present study showed that MAP3K2 overexpression resulted in decreased proliferation, as demonstrated by the CCK-8 assay (Fig. 5). To exclude the possibility that the decreased cell proliferation in MAP3K2-transfected cells resulted from cell apoptosis, we investigated whether MAP3K2 induces cell apoptosis. The Annexin V analysis showed that there was no significant difference in the percentage of apoptotic cells (Q2 + Q4) between the cells transfected with either the pCMV-HA-MAP3K2 or the pCMV-HA vector at 48 h after transfection (Fig. 6). The percentage of apoptotic cells in the DF1 cells transfected with pCMV-HA-MAP3K2 was 9.5% ± 0.7%, compared with the 8.8% ± 1.1% apoptotic cells in the DF1 cells transfected with the pCMV-HA vector. The percentage of apoptotic cells in ICPA-1 cells transfected with pCMV-HA-MAP3K2 was 11.2% ± 1.3% compared with the 11.2% ± 1.0% apoptotic cells in the ICPA-1 cells transfected with the pCMV-HA vector (Fig. 6a, b). These results suggest that the overexpression of MAP3K2 inhibits chicken cell proliferation but does not induce apoptosis.

### MAP3K2 overexpression attenuates the promotive effect of miR-17-92 cluster on DF1 cell proliferation

**Figure 7:**
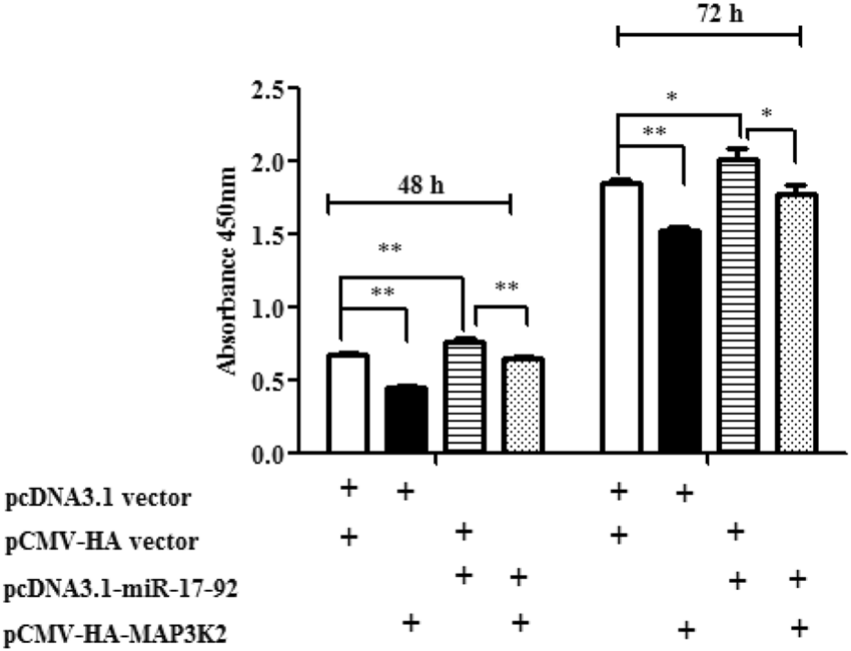
MAP3K2 overexpression attenuates the promotive effect of the miR-17-92 cluster on the proliferation of DF1 cells. DF1 cells were cotransfected with the designated plasmids, and cell proliferation was assayed at 48 and 72 h after transfection using the CCK-8 kit. “+” Denotes DF1 cells transfected with the designated plasmid. All data are representative of three independent experiments and shown as the mean ± SEM, *p < 0.05; **p < 0.01; determined by two-tailed Student’s t-test.

Based on the above results, we hypothesized that the miR-17-92 cluster potentially promotes chicken cell proliferation in part by suppressing MAP3K2 expression. To examine this hypothesis, DF1 cells were cotransfected with the pcDNA3.1-miR-17-92 cluster or the empty pcDNA3.1 vector and either pCMV-HA-MAP3K2 or pCMV-HA vector, and cell proliferation was assayed using the CCK-8 kit. The results of the CCK-8 assay showed that, as expected, compared with the negative control (co-transfection of pcDNA3.1 vector and pCMV-HA vector), the cotransfection of the pcDNA3.1 vector and pCMV-HA-MAP3K2 decreased the proliferation of DF1 cells at 48 (*p* < 0.01) and 72 h (*p* < 0.01), and co-transfection with the pCMV-HA vector and the pcDNA3.1-miR-17-92 cluster promoted cell proliferation at 48 (*p* < 0.01) and 72 h (*p* < 0.05). Interestingly, the cotransfection of the pcDNA3.1-miR-17-92 cluster and pCMV-HA-MAP3K2 reduced cell proliferation by 14% (*p* < 0.01) and 11.6% (*p* < 0.05), respectively, at 48 and 72 h, compared with the co-transfection of the pcDNA3.1-miR-17-92 cluster and the pCMV-HA vector, suggesting that MAP3K2 at least partially mediates the promotive effect of the miR-17-92 cluster on chicken cell proliferation (Fig. 7).

### MiR-17-92 cluster overexpression and MAP3K2 knockdown increase while MAP3K2 overexpression decreases c-Myc expression

**Figure 8:**
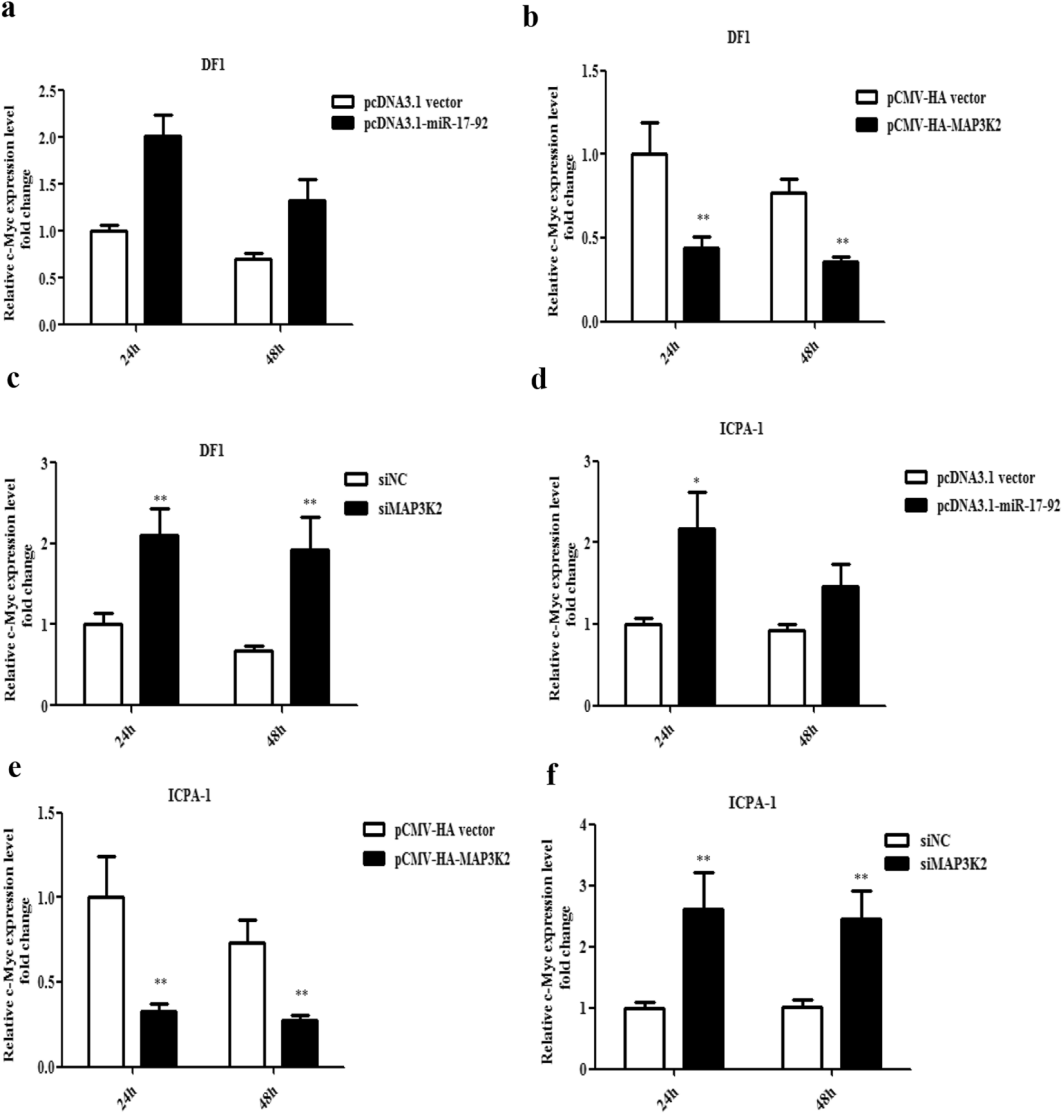
Effects of overexpression of miR-17-92 cluster and MAP3K2 and knockdown of MAP3K2 on c-Myc expression in DF1 and ICPA-1 cells. DF1 cells (**a**–**c**) and ICPA-1 cells (**d**–**f**) were transfected with the designated plasmids or siRNAs; at 24 and 48 h after transfection, total RNA was isolated, and c-Myc expression was detected using qRT-PCR. Gene expression was normalized to NONO mRNA level. Fold change is relative to either pcDNA3.1 vector, pCMV-HA vector or siNC at 24 h after transfection. All data are representative of three independent experiments and shown as the mean ± SEM. *p < 0.05; **p < 0.01; determined by two-tailed Student’s t-test.

MAPK signalling modules includes ERK1/2, ERK5, p38 and JNK and have many downstream effectors. To obtain insight into the mechanisms underlying the promotive effect of the miR-17-92 cluster on chicken cell proliferation, we performed the qRT-PCR analysis of the expression of four downstream effectors of the MAPK signalling pathway. The results showed that both the overexpression of the miR-17-92 cluster and the knockdown of MAP3K2 by siMAP3K2 significantly increased c-Myc expression in DF1 and ICPA-1 cells (Fig. 8a, c, d, f and Supplementary Fig. S3), but MAP3K2 overexpression significantly decreased c-Myc expression (Fig. 8b, e). In contrast, no obvious expression changes were observed for the other three downstream genes (Bax, Fas and TNF-α) (Supplementary Fig. S4–6). These data suggest that the miR-17-92 cluster targets MAP3K2, leading to the upregulation of c-Myc expression.

### C-Myc promotes cell proliferation

**Figure 9:**
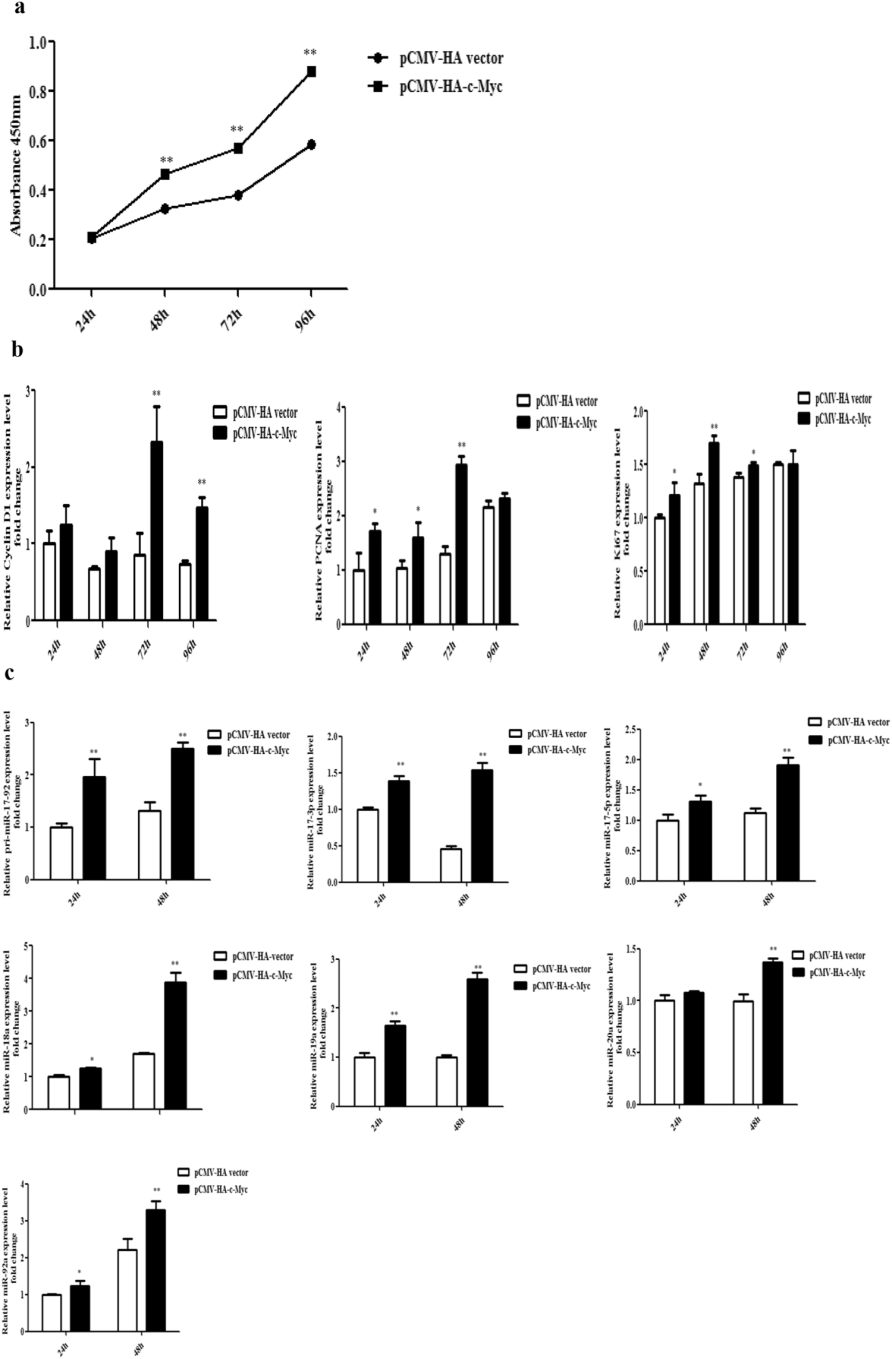
C-Myc overexpression promotes chicken cell proliferation and miR-17-92 cluster expression. (**a**) Effect of c-Myc overexpression on the proliferation of DF1 cells. Cells were transfected with either pCMV-HA-c-Myc or pCMV-HA vector, and cell proliferation was assayed at the designated time points after transfection using the CCK-8 kit. (**b**) qRT-PCR analysis of PCNA, Cyclin D1 and Ki67 in DF1 cells. Cells were transfected with pCMV-HA-c-Myc or pCMV-HA vector at the designated time points after transfection, and total RNA was isolated and gene expression was assessed using qRT-PCR. Gene expression was normalized to NONO mRNA level. Fold change is relative to pCMV-HA vector at 24 h after transfection. (**c**) DF1 cells were transfected with either pCMV-HA-c-Myc or pCMV-HA vector at the designated time points after transfection, total RNA was isolated and the expression of pri-miR-17-92 and its mature miRNA members was quantified using qRT-PCR and stem-loop qRT-PCR, respectively. The pri-miR-17 -92 expression was normalized to NONO mRNA level, and the mature miRNA expression was normalized to U6 snRNA expression level. Fold change is relative to pCMV-HA vector at 24 h after transfection. All data are representative of three independent experiments and shown as the mean ± SEM. *p < 0.05; **p < 0.01; determined by two-tailed Student’s t-test.

C-Myc plays a central role in cell proliferation^35^. The results of the present study showed that the miR-17-92 cluster promoted cell proliferation and the miR-17-92 cluster promoted c-Myc expression by downregulating MAP3K2 (Fig. 9). These results suggested that c-Myc might mediate the promotive effect of miR-17-92 on chicken cell proliferation. To verify this mechanism, we investigated the effect of c-Myc overexpression on chicken cell proliferation using the CCK-8 kit. The CCK-8 assay results showed that, c-Myc overexpression strikingly promoted chicken cell proliferation in DF1 cells (Fig. 9a). Consistently, the gene expression of proliferation markers (PCNA, Cyclin D1 and Ki67) was elevated by c-Myc overexpression (Fig. 9b). Collectively, these results suggest the miR-17-92 cluster promotes chicken cell proliferation at least in part by upregulation of c-Myc expression.

C-Myc directly regulates human miR-17-92 cluster expression^36,37^. However, it is not clear whether c-Myc regulates chicken miR-17-92 cluster expression. We investigated the effect of c-Myc overexpression on miR-17-92 cluster expression in DF1 cells. The gene expression results showed that, compared with pCMV-HA vector, c-Myc overexpression increased the expression of the pri-miR-17-92 cluster transcript and mature miR-17-3p, miR-17-5p, miR-18a, miR-19a, miR-20a and miR-92a, suggesting that c-Myc regulates chicken miR-17-92 cluster expression (Fig. 9c).

## Discussion

Here, we demonstrated that overexpression of the miR-17-92 cluster promotes chicken cell proliferation at least in part by downregulating MAP3K2, and c-Myc is at least a key downstream effector of the miR-17-92 cluster in promoting chicken cell proliferation. The miR-17-92 cluster promotes cell proliferation in various mammalian cell types^10,38^. Several members of the miR-17-92 cluster regulate cell proliferation. For example, miR-17-5p promoted cell proliferation in gastric^39^ and colorectal cancer cell lines^40^, and miR-20a promoted cell proliferation in human lung cancer^41^ and multiple myeloma^42^. In the present study, we demonstrated that the miR-17-92 cluster promotes chicken cell proliferation through its two members, miR-17-5p and miR-20a (Fig. 2).

MAP3K2 is an important component of the MAPK signalling pathway, which converts extracellular stimuli into cell proliferation. Several miRNAs have been identified to target MAP3K2. For example, miR-520b suppressed tumour formation in breast cancer and hepatocellular carcinoma by targeting MAP3K2 and cyclin D1, miR17/20a inhibited tumour growth via targeting the MAP3K2-Erk5 pathway *in vivo* ^43^, and miR-26a promoted glioblastoma cell growth *in vitro* via targeting MAP3K2^30^. In the present study, we demonstrated that miR-17-5p/20a regulates chicken cell proliferation by targeting chicken MAP3K2 (Fig. 2 and 4). Previous studies have shown that MAP3K2 mediates cell proliferation. Knockdown of MAP3K2 using RNA interference inhibited the growth of hepatocarcinoma cells and lung cancer cells^27,34^, whereas knockdown of MAP3K2 promoted the proliferation of HeLa cells^44^. The results of the present study demonstrated that MAP3K2 overexpression inhibited the proliferation of DF1 and ICPA-1 cells (Fig. 5). These data suggest that the roles of MAP3K2 in cell proliferation differ dependent on cell types and cellular context.

C-Myc regulates a number of key normal cellular processes such as growth, proliferation and apoptosis, in mammals^45,46^ and birds^47,48^. In addition, c-Myc also plays important roles in tumourigenesis, tumour maintenance and metastasis. To further understand the mechanism underlying the promotive effect of the miR-17-92 cluster on cell proliferation, we examined the expression of downstream effectors of the MAPK signalling pathway. These results provided the first evidence that miR-17-92 cluster overexpression increased c-Myc gene expression (Fig. 8a, d), and further analysis showed that c-Myc overexpression promoted chicken cell proliferation (Fig. 9), consistent with its role in mammalian cell proliferation. Taken together, these data suggest that c-Myc is a key downstream effector mediating the promotive effect of miR-17-92 cluster, which is supported by previous reports showing that NFATC1 promotes proliferation by upregulating c-Myc through the activation of the MAPK signalling pathway^49^, and DAPK3 controls proliferation through the activation of MAPK/ERK/c-Myc signalling in A549 cells^50^.

Several members of the miR-17-92 cluster target the MAPK signalling pathway. For example, miR-17 and miR-19a directly target MAPK1^20^, miR-17-5p can activate p38 MAPK-HSP27 signalling^51^, and miR-20a-5p can activate MAPK/ERK and cAMP/PKA signalling pathways^52^. In addition, bioinformatics analysis showed that miR-19a targets K-RAS and RAF1, two principal components of the MAPK signalling pathway^20^. In the present study, we provided evidence that two members of miR-17-92 cluster, miR-17-5p and miR-20a, target the MAPK signalling pathway in chicken cells.

The ERK5 MAPK signalling module plays an important role in the regulation of cell proliferation and cell differentiation^53^. MAP3K2 is an upstream kinase of the ERK5 MAPK signalling module^54^. The c-Myc is one of the downstream effectors of the ERK5 MAPK signalling module^55^. In the present study, we demonstrated that the miR-17-92 cluster promoted chicken cell proliferation by targeting MAP3K2, accompanied by increased c-Myc expression, and c-Myc overexpression promoted chicken cell proliferation. Taken together^54,55^, these results show that the miR-17-92 cluster promotes chicken cell proliferation, at least in part via targeting of the ERK5 MAPK signalling module.

**Figure 10:**
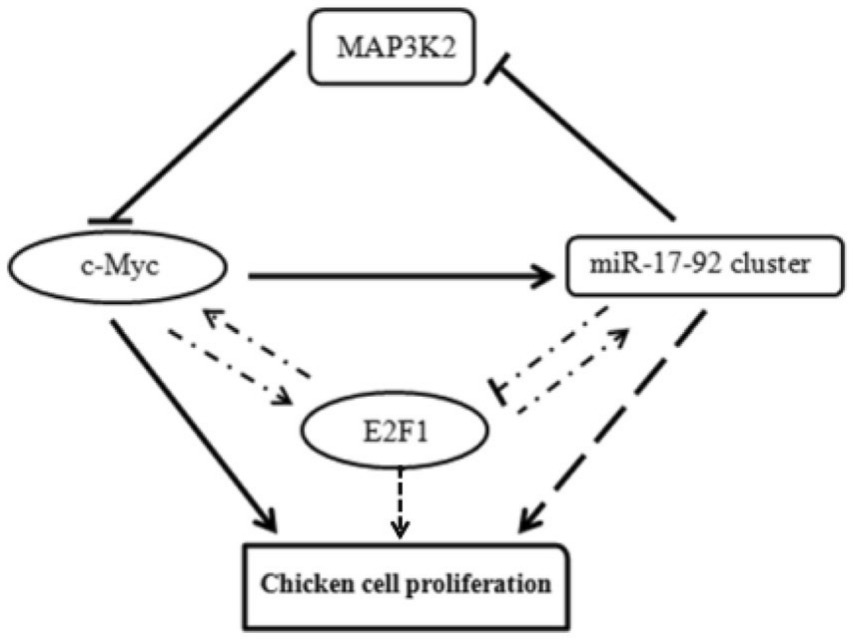
Model of the proposed regulatory network of chicken cell proliferation. The miR-17-92 cluster, MAP3K2, E2F1 and c-Myc form a complex regulatory feedback network to regulate chicken cell proliferation. The miR-17-92 cluster promotes chicken cell proliferation via upregulation of c-Myc by targeting MAP3K2, which inhibits c-Myc expression through the MAPK signaling pathway. In addition to MAP3K2, the miR-17-92 cluster can also promote cell proliferation via targeting other target genes such as PTEN and TGFBR2. The c-Myc and E2F1 can activate the transcription of each other, and both of them directly promote the miR-17-92 cluster expression. However, the miR-17-92 cluster postranscriptionally suppresses E2F1 expression through its two members (miR-17-5p and miR-20a). thus counterbalancing the positive feedback loop of E2F1 and c-Myc. Darker lines denote evidence provided in the present study, and the dashed lines denote evidence based on the literature [17, 58].

In the present study, the miR-17-92 cluster increased c-Myc expression through the downregulation of MAP3K2, and increased c-Myc expression resulted in the promotion of chicken cell proliferation. However, it has been demonstrated that c-Myc transcriptionally activates miR-17-92 cluster expression in mammals^16,56^. We recently closed the genomic gap upstream of the chicken miR-17-92 cluster, and consistently identified a conserved c-Myc binding site located in the chicken MIR17HG promoter region^57^. In agreement with the mammalian studies in the present study, we demonstrated that c-Myc overexpression upregulated miR-17-92 cluster expression (Fig. 9c). These data suggest c-Myc also transcriptionally activates chicken miR-17-92 cluster expression. C-Myc and E2F1 are vital regulators of cell cycle progression and proliferation. Both of them can activate the transcription of each other^16^ and transcriptionally activate miR-17-92 cluster expression by directly binding to the MIR17HG promoter^17,58^. However, two members of the miR-17-92 cluster (miR-17 and miR-20a) can posttranscriptionally downregulate E2F1 expression^11^, thus counterbalancing the positive feedback loop of E2F1 and c-Myc. In the present study, we observed that the overexpression of the miR-17-92 cluster increased the expression of E2F1 (Fig. 2b,c). The increased E2F1 expression may reflect the combinatorial effects of the positive E2F1 regulator, c-Myc, whose expression was induced by miR-17-92 cluster overexpression, and the negative E2F1 regulator, miR-17/20a, encoded by the miR-17-92 cluster. Taken together^11,16,17^, these data suggest that the miR-17-92 cluster, c-Myc and E2F1 form a complex regulatory network in chicken cell proliferation (Fig. 10).

In the present study, we demonstrated that miR-17/20a mediates the promotive effect of the miR-17-92 cluster on cell proliferation. Given that the miR-17-92 cluster encodes at least six individual miRNAs, we cannot exclude the likelihood that other members of the miR-17-92 cluster also contribute to the promotive effect of the miR-17-92 cluster on chicken cell proliferation. In addition, several signalling pathways are targeted by the miR-17-92 cluster^20–22^; however, apart from the MAPK signalling pathway, we cannot rule out whether the other signalling pathways also contribute to the promotive effect of the miR-17-92 cluster. These questions should be addressed in the future.

## Materials and Methods

### Materials

The miRNA-17-5p inhibitor, miRNA-20a inhibitor and negative control were purchased from Ribobio (Guangzhou, China). SiMAP3K2 and siNC were obtained from GenePharma (Suzhou, China), and MAP3K2 antibody was obtained from Santa Cruz Biotechnology (Santa Cruz, CA, USA). The β-actin antibody was obtained from TransGene (Beijing, China). The psi-CHECK2 and pCMV-HA vectors were purchased from Promega (Madison, WI, USA). DF1 cells were purchased from the Institute of Biochemistry and Cell Biology, Chinese Academy of Sciences. Immortalized chicken preadipocytes (ICPA-1) were generated in the lab^59^.

### Cell culture

DF1 cells and ICPA-1 cells were cultured in Dulbecco’s modified Eagle’s medium (no pyruvate, high glucose formulation; Gibco-BRL) supplemented with 10% heat inactivated foetal bovine serum (Gibco, USA) at 37 °C in a humidified atmosphere of 5% CO_2_ in air, with media changes every other day.

### Isolation and Culture of Primary Chicken Preadipocytes

Primary chicken preadipocytes were isolated from 10-day-old Arbor Acres (AA) broilers and cultured as previously described^32^, 3–5 g of abdominal adipose tissue was collected, washed with prewarmed PBS, minced with scissors, and subsequently digested in 2 mg/mL collagenase type I (Invitrogen, USA) for 65 min at 37 °C with shaking. After digestion, the cell suspension was filtrated through a 20-µm sieve, and the filtrate was centrifuged at 200 g for 10 min at room temperature. The cell pellet comprised chicken stromal vascular cells (preadipocytes). The cell pellet was suspended and seeded at a density of 5 × 10^4^ cells/mL in DMEM/F12 medium (Gibco, USA) supplemented with 10% foetal calf serum (Gibco, USA) and maintained at 37 °C in a humidified atmosphere containing 5% CO_2_ in air.

### Plasmid construction

The 3′UTR (598 bp) of chicken MAP3K2 was amplified from chicken cDNA using the following two primers, forward: 5′- CCTCGAGGGCATTTATTTTCTATTTG-3′, which introduced an *Xho* I site, and reverse: 5′- TTGCGGCCGCAACAGTGTTGAGTTCTTTTC-3′, which introduced a *Not* I site. The RT-PCR product and psi-CHECK2 vector were double digested with *Xho* I and *Not* I, and ligated to yield the MAP3K2 3′UTR reporter psi-CHECK2-MAP3K2-3′UTR-WT. In addition, the MAP3K2 3′UTR mutant reporter psi-CHECK2-MAP3K2-3′UTR-MUT was also generated using gene synthesis by Invitrogen, in which the sequence GUGA was mutated to ACUG within both the predicted miR-17-5p and miR-20a binding sites, respectively. For the chicken miR-17-92 cluster expression vector pcDNA3.1-miR-17-92 cluster, an 879-bp genomic DNA fragment encoding the mir-17-92 cluster precursor was amplified from chicken genomic DNA using PCR with the forward primer: 5′-GGTACCTTCTTTTCTTTCAGCAGG-3′, which introduced a *Kpn* I site, and the reverse primer: 5′-CCTCGAGGTGTTTCAGCCTCTATCCC-3′, which introduced a *Xho* I site. The resulting PCR product was double digested with *Kpn* I and *Xho* I and cloned between the *Kpn* I and *Xho* I sites of pcDNA3.1(+) (Invitrogen, USA). The pcDNA3.1-miR-17-92 cluster construct was sequenced and its correct expression was confirmed using stem-loop qRT-PCR (Supplementary Fig. S7). For the MAP3K2 expression vector pCMV-HA-MAP3K2, the full-length coding sequence of MAP3K2 was amplified from chicken pooled cDNA using RT-PCR with the following specific primers, forward: 5′- CGTCGACGTTCTTCAGTGTGAAATGA-3′, which introduced a *Sal* I site, and reverse: 5′- GGTACCTGCAGTCAAATAGAAAATAAATG -5′, which introduced a *Kpn* I site. The resulting PCR product and pCMV-HA expression vector were double digested with *Sal* I and *Kpn* I and then ligated to generate pCMV-HA-MAP3K2. The pCMV-HA-MAP3K2 was sequenced, and the correct expression was validated by western blotting (Supplementary Fig. S8). For the c-Myc expression vector pCMV-HA-c-Myc, the full-length c-Myc coding sequence was amplified from chicken pooled cDNA using RT-PCR with the following specific primers: 5′-GGAATTCGCCTCCCCAGCAAGAACTA -5′, which introduced an *EcoR* I site, and 5′-CCTCGAGGCACGAGAGTTCCTTAGCTG -5′, which introduced an *Xho* I site. The PCR product and pCMV-HA-vector were double digested with EcoR I and Xho I and ligated to yield pCMV-HA-c-Myc. The pCMV-HA-c-Myc was sequenced and its correct expression was confirmed by western blotting using an anti-HA tag antibody (Supplementary Fig. S9).

### MiRNA and siRNA transfection

DF1 cells were seeded at a density of 5 × 10^4^ cells/mL on to 24-well plates. At 30% confluency, the cells were respectively transfected with the miRNA-17-5p inhibitor, miRNA-20a inhibitor, miRNA negative control (miR-NC), siMAP3K2 and its corresponding negative controls (siNC) using Lipofectamine 2000 (Invitrogen, Life Technologies, USA) according to the manufacturer’s instructions. The following siRNA sequences were used: siNC sense (5′-3′) UUCUCCGAACGUGUCACGUTT, antisense (5′-3′) ACGUGACACGUUCGGAGAATT, siMAP3K2 sense (5′-3′) CCAGAUAACCAUCAGGAAUTT, and siMAP3K2 antisense (5′-3′) AUUCCUGAUGGUUAUCUGGTT. At 48 h post transfection, total RNAs were extracted, and MAP3K2 gene expression was analysed using qRT-PCR.

### RNA Extraction and qRT-PCR

Total RNA was extracted from cells using TRIzol reagent (Invitrogen) according to the manufacturer’s instructions. For gene expression analysis, a total of 1 μg of RNA for each sample was reverse-transcribed to cDNA using Reverse Transcription kits (Promega, USA), and qRT-PCR was performed on a 7500 real-time PCR System (Applied Biosystem) using FastStart Universal SYBR Green Master [Rox] (Roche, USA). The primers used in the present study are shown in Table 1. NONO was used as the internal reference for normalization^60,61^. The relative mRNA expression was analysed using the 2^–ΔΔCt^ method^62^.

**Table 1:** Primers used for qRT-PCR gene expression analysis.

Gene name	Forward/reverse primers (5′-3′)
Ki67	F: AGGATGGAAGCAAGTCACCTGGAT R: CTTCTGAACGGGGACTGGAATCTT
PCNA	F: CGTCTCATGTCTCCTTGGTGCA R: GGACATGCTGGTGAGGTTCA
Cyclin D1	F: CTCGGAGCTACCTGCATGTTTGT R: TTTACGGATGATCTGTTTGGTGT
E2F1	F: CCCCGCACCTCCTCATCGTCT R: CAAGTTCAGCTTCCGCTTCACCG
NONO	F: AGAAGCAGCAGCAAGAAC R: TCCTCCATCCTCCTCAGT
Pri-miR-17-92	F: CATCTACTGCCCTAAGTGCTCCTT R: GCTTGGCTTGAATTATTGGATGA

For miRNA expression analysis, miRNAs were reverse transcribed from total RNA using a reverse transcription system kit (Promega, USA) with the designed miRNA-specific stem loop-RT primers according to the manufacturer’s instructions. Real-time PCR was performed using FastStart Universal SYBR Green Master [Rox] (Roche, USA) with miRNA-specific stem loop-RT primer and universal primer (URP). The miRNA-specific stem loop-RT primers and universal primers are listed in Table 2. Real-time PCR was performed on a 7500 real-time PCR System (Applied Biosystem), and the PCR reactions were conducted at 50 °C for 2 min and 95 °C for 10 min, followed by 40 cycles at 95 °C for 15 s and 60 °C for 60 s using an ABI 7500 fast real-time PCR system. U6 snRNA was used as an internal control, and the relative miRNA expression was analysed using the 2^−ΔΔCt^ method^62^.

**Table 2:** Primers used for stem-loop qRT-PCR analysis.

Primer name	Primer sequences (5′-3′)	Tm (°C)
miR-17-5p-F1	ACACTCCAGCTGGGCAAAGTGCTTACAGTGCA	55
miR-17-5p-R1	CTCAACTGGTGTCGTGGAGTCGGCAATTCAGTTGAGACTACCTG	
miR-17-3p-F1	ACACTCCAGCTGGGACTGCAGTGAAGGC	55
miR-17-3p-R1	CTCAACTGGTGTCGTGGAGTCGGCAATTCAGTTGAGACAAGTGC	
miR-18a-F1	ACACTCCAGCTGGGTAAGGTGCATCTAGTG	55
miR-18a-R1	CTCAACTGGTGTCGTGGAGTCGGCAATTCAGTTGAGTATCTGCA	
miR-19a-F1	ACACTCCAGCTGGGTGTGCAAATCTATGCAA	55
miR-19a-R1	CTCAACTGGTGTCGTGGAGTCGGCAATTCAGTTGAGTCAGTTTT	
miR-20a-F1	ACACTCCAGCTGGG TAAAGTGCTTATAGTGC	55
miR-20a-R1	CTCAACTGGTGTCGTGGAGTCGGCAATTCAGTTGAGCTACCTGC	
miR-92-F1	ACACTCCAGCTGGGTATTGCACTTGTCCC	55
miR-92-R1	CTCAACTGGTGTCGTGGAGTCGGCAATTCAGTTGAGCAGGCCGG	
U6-F	GCGCGTCGTGAAGCGTTC	55
U6-R	GTCGTATCCAGTGCAGGGTCCGAGGTATTCGCACTGGATACGACAAATA	
URP	GTGCAGGGTCCGAGGT	

### Luciferase reporter gene assay

DF1 cells were grown at approximately 30~50% confluence, transfected with either psiCHECK2-MAP3K2-3′UTR-WT or psi-CHECK2-MAP3K2-3′UTR-MUT and designated plasmids or miRNA inhibitors in 24-well plates using Lipofectamine 2000 (Invitrogen, USA). At 48 h post transfection, luciferase activity was measured using a dual luciferase assay system (Promega, USA) according to the manufacturer’s instructions.

### Cell proliferation assay

Cell proliferation was determined using the Cell Counting Kit-8 (CCK-8) (DOJINDO, Japan) according to the manufacturer’s instructions. The cells were seeded onto 24-well plates at 1 × 10^4^ cells/well. After 6 h of culture, the cells were transfected with the designated miRNA inhibitors or plasmids. At the designated time points, 50 μL of CCK-8 solution was added to each well, and the cells were incubated for an additional 3 h. The absorbance was determined at 450 nm. All experiments were repeated at least three times.

### Immunoprecipitation (IP)

DF1 cells were with the indicated miRNA inhibitors. At 48 h after transfection, the cells were washed three times with PBS, and lysed on ice for 30 min using RIPA buffer containing PMSF (RIPA:PMSF = 100:1), with periodic mixing, followed by centrifugation at 12,000 × g for 10 min at 4 °C to remove the cell debris. Subsequently, 3 μL of MAP3K2 antibody was added to 200 μL of cell lysate, and the mixture was incubated at 4 °C overnight with rotary agitation. After incubation, 20 μL of protein A/G sepharose beads (Santa, USA) was added, and the mixture was incubated for an additional 4 h at 4 °C with rotary agitation. After washing the beads three times with RIPA buffer, the denaturing loading buffer (5×) was added to the beads and boiled for 5 min at 100 °C.

### Western blot analysis

Protein samples were subjected to 10% SDS-PAGE separation. The proteins were transferred to PVDF microporous membranes (Millipore, USA), and the blots were probed with primary antibodies against MAP3K2 (1:500) and β-actin (1:1000). β-actin was used as a loading control. Immunoreactive bands were visualized using an ECL plus detection kit (Beyotime, China), and the images were captured using a Bio-Rad camera system (Bio-Rad, USA).

### Annexin V/propidium iodide (AV-PI) double staining and flow cytometry

DF1 cells were transfected with either pCMV-HA-MAP3K2 or the pCMV-HA vector. At 48 h after transfection, the cells were trypsinized and filtered through a 70-µm filter to yield single cell suspensions. Each single cell suspension was washed twice with cold PBS. After washing, the cells were incubated with 200 μL of dye liquor containing 1 mL of Binding Buffer and 50 μL of Annexin V, and maintained under cold conditions for 30 min. Subsequently, 3 μL of propidium iodide (PI) and 300 μL of PBS were added. The cells were analysed using flow cytometry on a FACScan (Becton Dickinson, USA). In the scatter diagram, the right upper quadrant and right lower quadrant represent the number of apoptotic cells and the left lower quadrant represents the number of normal cells. The apoptosis ratio is the number of apoptotic cells (right upper quadrant plus right lower quadrant)/total cells. The experiment was repeated three times.

### Statistical analysis

The results are presented as the mean ± SEM. Two-tailed Student’s t-test was performed to compare the differences between experimental and control groups. The differences were considered significant at *p < 0.05 and highly significant at **p < 0.01.

## Electronic supplementary material

Supplementary Information
