# Supplementary material for: MiR-17-5p and miR-20a promote chicken cell proliferation at least in part by upregulation of c-Myc via MAP3K2 targeting
Source: Sci Rep. 2017 Nov 20;7:15852. doi: 10.1038/s41598-017-15626-9 (PMC5696470; doi:10.1038/s41598-017-15626-9)
Supplement: Supplementary file 1 — Supplementary Information [file 41598_2017_15626_MOESM1_ESM.pdf]

MiR-17-5p and miR-20a promote chicken cell proliferation at least in part by upregulation of c-Myc via MAP3K2 targeting

Xiaofei Zhang[1,2,3], He Song[1,2,3], Shupei Qiao[1,2,3], Jing Liu[1,2,3], Tianyu Xing[1,2,3], Xiaohong Yan[1,2,3], Hui Li[1,2,3], Ning Wang [1,2,3*]

[1]Key Laboratory of Chicken Genetics and Breeding, Ministry of Agriculture, Harbin 150030, Heilongjiang, China
[2]Key Laboratory of Animal Genetics, Breeding and Reproduction, Education Department of Heilongjiang Province, Harbin 150030, Heilongjiang, China
[3]Key Laboratory of Animal cells and genetic engineering of Heilongjiang Province, Harbin 150030, Heilongjiang, China

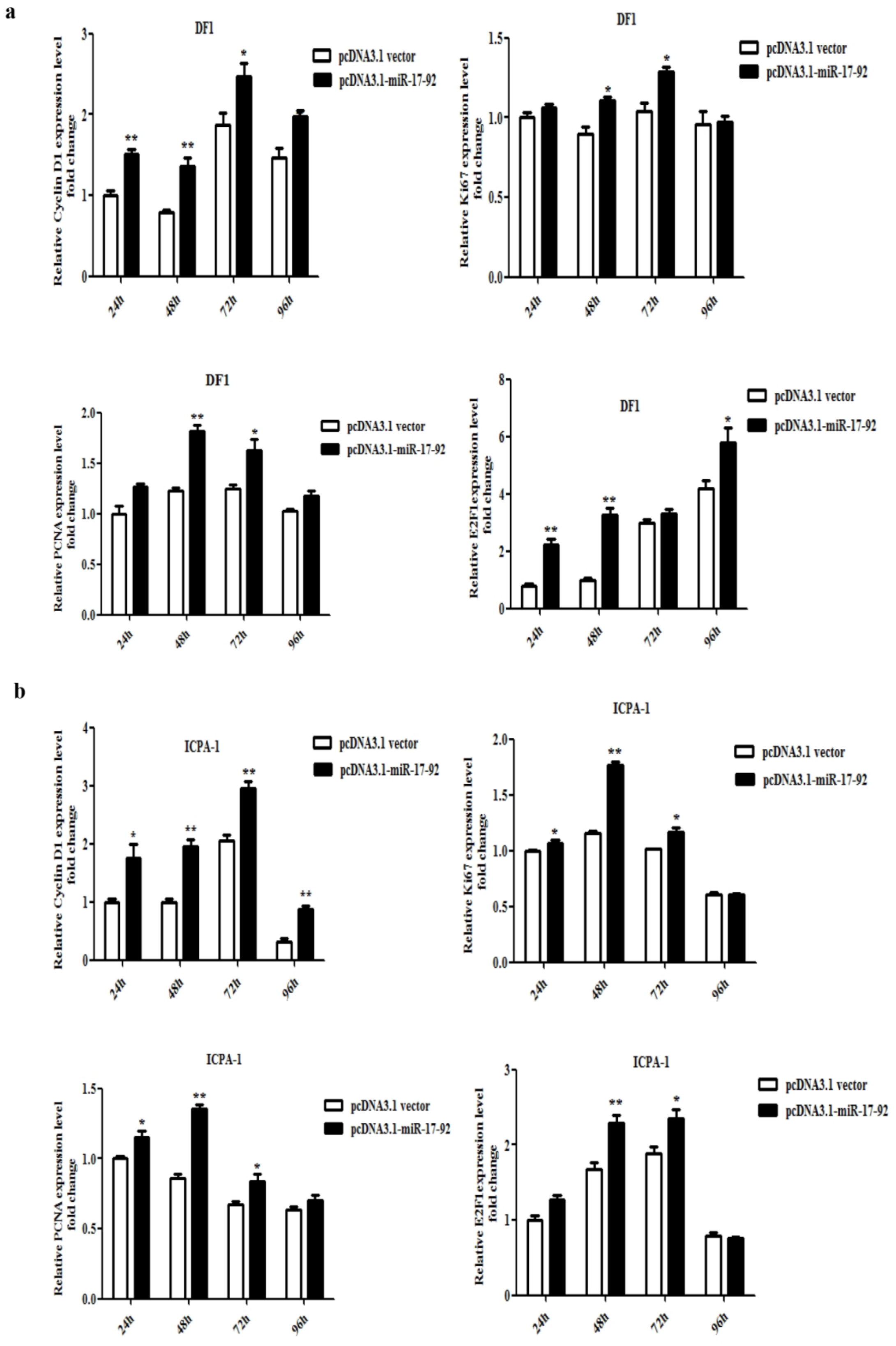
a
DF1
Relative Cyclin D1 expression level fold change
Relative Ki67 expression level fold change
Relative PCNA expression level fold change
Relative E2F1expression level fold change
pcDNA3.1 vector
pcDNA3.1-miR-17-92
24h
48h
72h
96h
b
ICPA-1

Supplementary Figure S1. Effect of miR-17-92 cluster overexpression on the expression of proliferation marker genes in DF1 cells and ICPA-I cells. (**a** and **b**) Cells were transfected with either pcDNA3.1-miR-17-92 or pcDNA3.1 vector, total RNA was subsequently isolated at the designated time points, and the gene expression of Cyclin D1, Ki67, PCNA and E2F1 was assessed at the designated time points using qRT-PCR. Gene expression was normalized to NONO mRNA level. Fold change is relative to pcDNA3.1 vector at 24 h after transfection. All data are representative of three independent experiments and shown as the mean ± SEM. * $p < 0.05$; ** $p < 0.01$; determined by two-tailed Student's t-test.

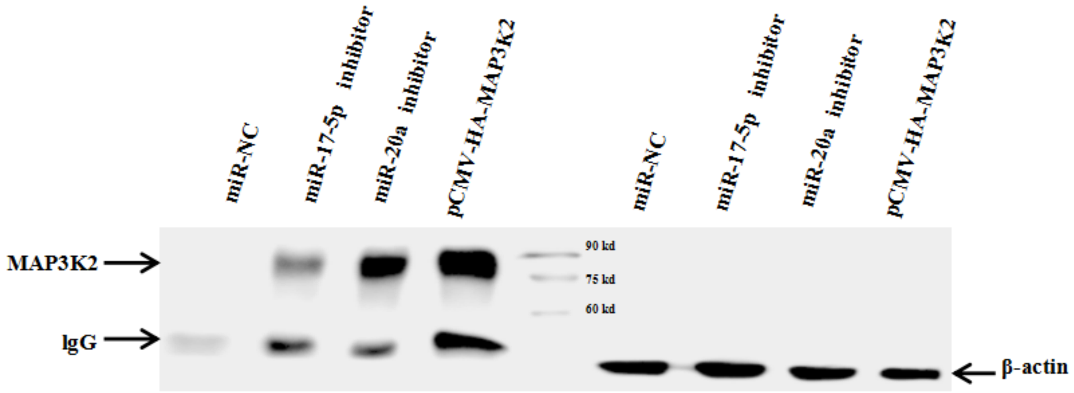

Supplementary Figure S2. IP-western blot analysis of MAP3K2 in DF1 cells. DF1 cells were transfected with the indicated miRNA inhibitors and pCMV-HA-MAP3K2 (positive control). At 48 h post transfection, the cells were harvested, and MAP3K2 protein expression was assessed using IP-western blot analysis (left panel). Matched inputs were assayed for β-actin using western blotting (right panel).

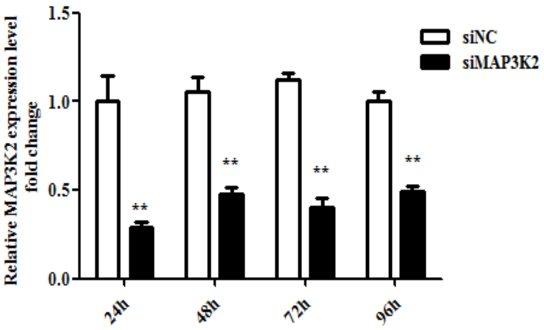

Supplementary Figure S3. The efficiency of the knockdown effect of MAP3K2 siRNA (siMAP3K2). DF1 cells were transfected with siMAP3K2. At 24, 48, 72 and 96 h after transfection, total RNA was isolated, and MAP3K2 expression was detected using qRT-PCR. The NONO gene was used as an internal control. Gene expression was normalized to NONO mRNA level. Fold change is relative to siNC at 24 h after tranfection. All data are representative of three independent experiments and shown as the mean ± SEM. ** $p < 0.01$; determined by two-tailed Student's t-test.

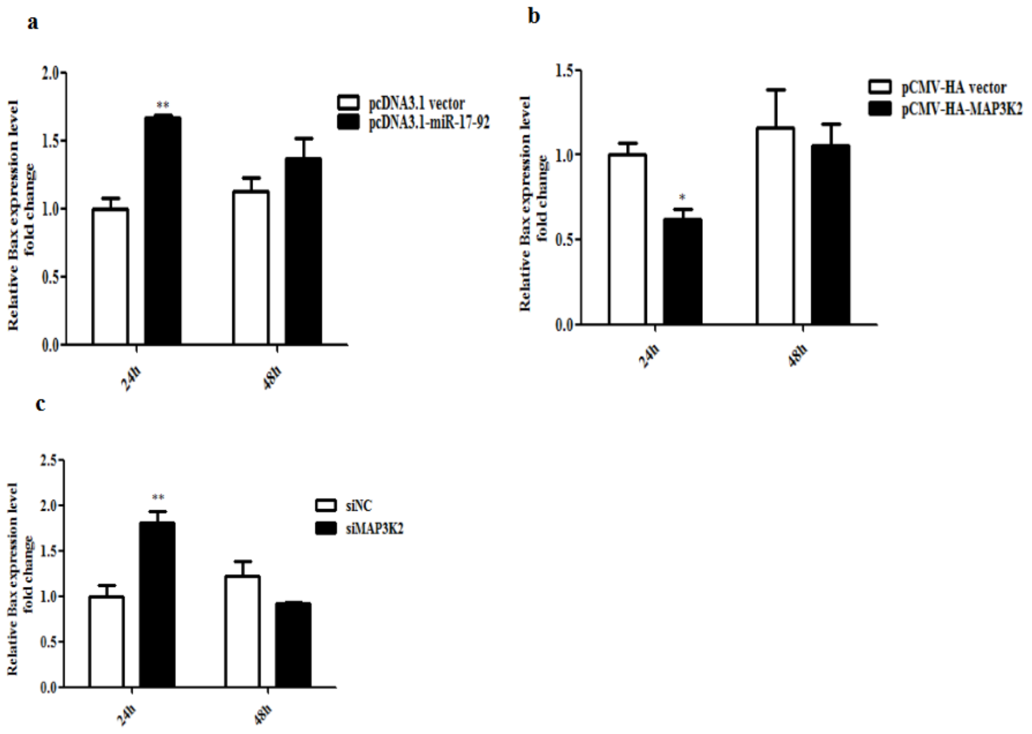

Supplemental Figure S4. Effects of overexpression of miR-17-92 cluster and MAP3K2 and knockdown of MAP3K2 on Bax expression. DF1 cells (**a**-**c**) were transfected with designated plasmids or siRNAs. At 24 and 48 h, respectively, after transfection, total RNA was isolated, and Bax expression was detected using qRT-PCR. Gene expression was normalized to NONO mRNA level. Fold change is relative to either pcDNA3.1 vector, pCMV-HA vector or siNC at 24 h after transfection. All data are representative of three independent experiments and shown as the mean ± SEM. * $p < 0.05$; ** $p < 0.01$; determined by two-tailed Student's t-test.

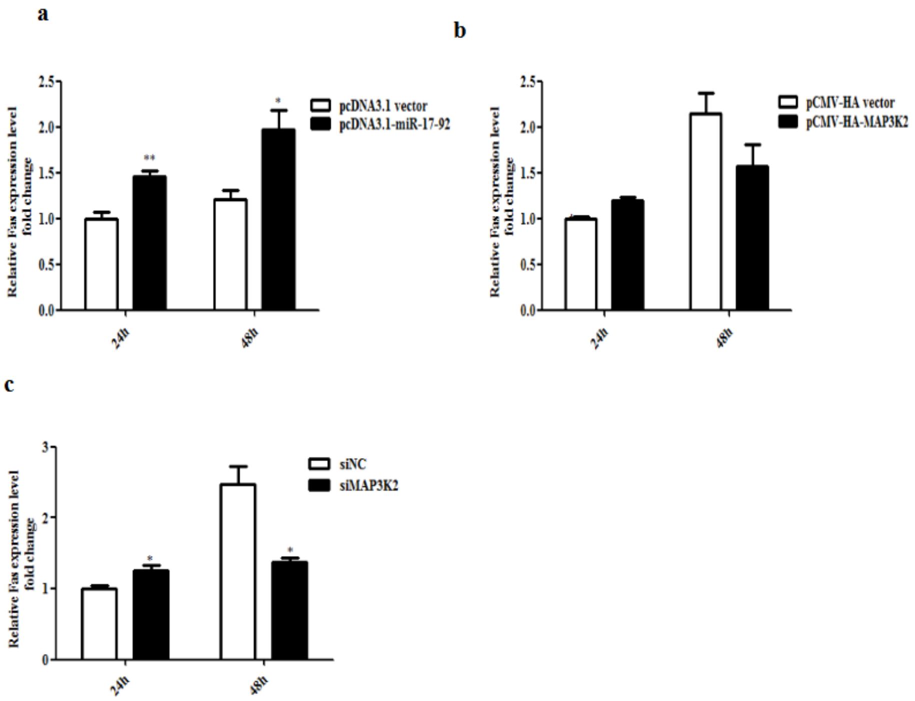

Supplemental Figure S5. Effects of the overexpression of the miR-17-92 cluster and MAP3K2 and the knockdown of MAP3K2 on Fas~~AS~~ expression in DF1 cells. DF1 cells (**a-c**) were transfected with designated plasmids or siRNAs. At 24 and 48 h, respectively, after transfection, total RNA was isolated, and Fas expression was detected using qRT-PCR. Gene expression was normalized to NONO mRNA level. Fold change is relative to either pcDNA3.1 vector, pCMV-HA vector or siNC at 24 h after transfection. All data are representative of three independent experiments and shown as the mean ± SEM. * $p < 0.05$; ** $p < 0.01$; determined by two-tailed Student's t-test.

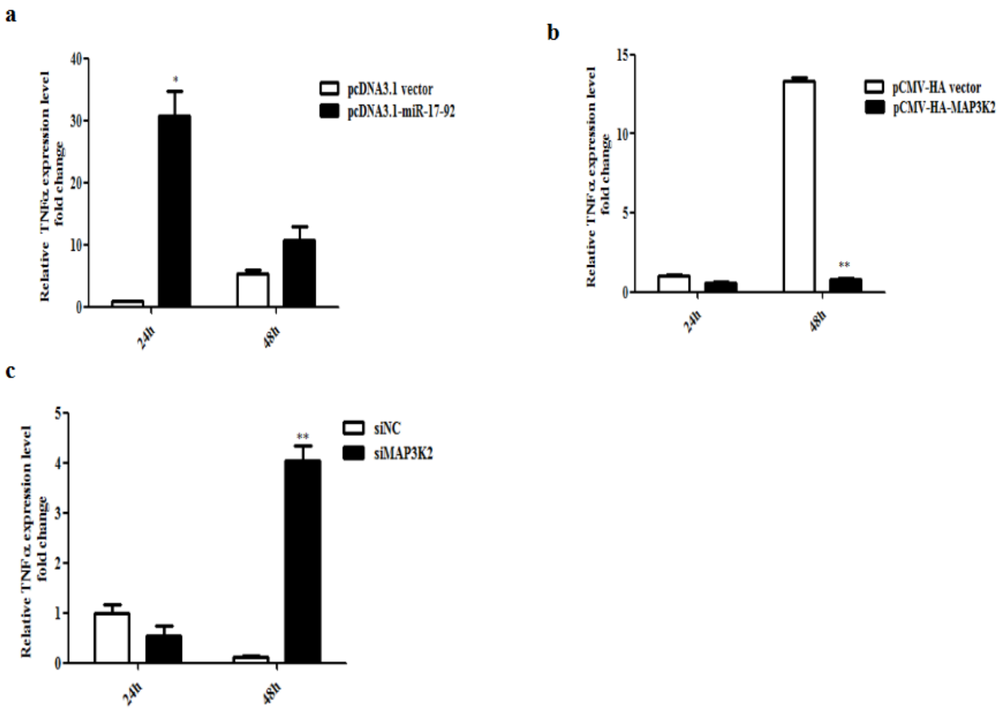

Supplemental Figure S6. Effects of overexpression of miR-17-92 cluster and MAP3K2 and knockdown of MAP3K2 on TNFα expression in DF1 cells. DF1 cells (**a-c**) were transfected with designated plasmids or siRNAs. At 24 and 48 h, respectively, after transfection, total RNAs were isolated, and TNFα expression was detected using qRT-PCR. Gene expression was normalized to NONO mRNA level. Fold change is relative to either pcDNA3.1 vector, pCMV-HA vector or siNC at 24 h after transfection. All data are representative of three independent experiments and shown as the mean ± SEM. * $p < 0.05$; ** $p < 0.01$; determined by two-tailed Student's t-test.

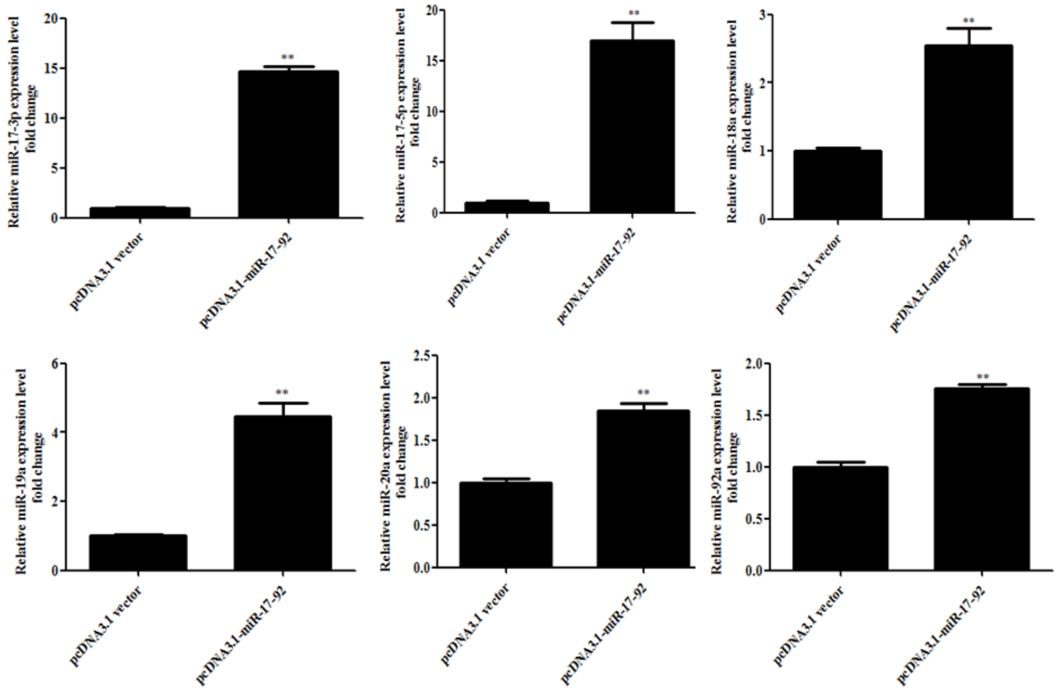

Supplementary Figure S7. The relative expression of individual members of miR-17-92 cluster in DF1 cells transfected with pcDNA3.1-miR-17-92. DF1 cells were transfected with either pcDNA3.1-miR-17-92 or pcDNA3.1 vector. At 24 h after transfection, total RNA was isolated; the relative miRNA expression was analysed using stem-loop qRT-PCR. Fold change is relative to pcDNA3.1 ~~pCMV-HA~~ vector after normalization to U6 snRNA. All data are representative of three independent experiments and shown as the mean ± SEM. ** $p < 0.01$; determined by two-tailed Student's t-test.

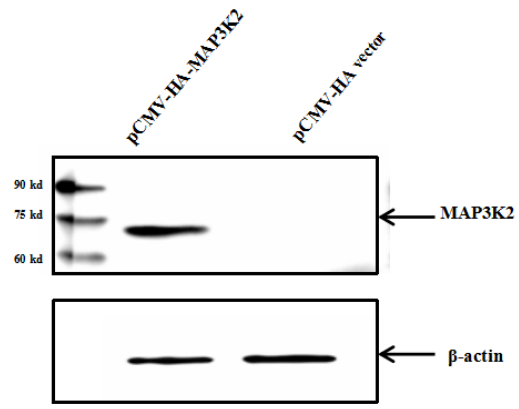

Supplementary Figure S8. Identification of MAP3K2 expression vector (pCMV-HA-MAP3K2) using western blotting. DF1 cells were transfected with either pCMV-HA-MAP3K2 or pCMV-HA vector. At 48 h post-transfection, the cells were harvested, and MAP3K2 protein was detected by western blotting using an anti-HA tag antibody (top panel). β-actin was used a loading control (bottom panel).

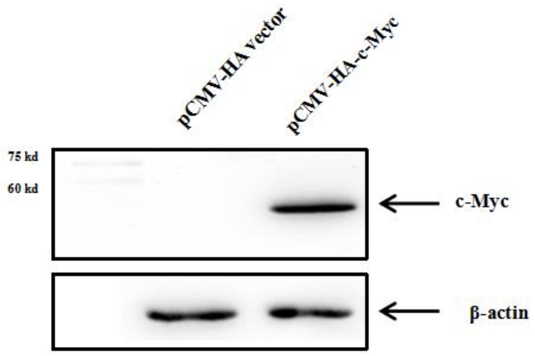

Supplementary Figure S9. Identification of c-Myc expression vector (pCMV-HA-c-Myc) using western blotting. DF1 cells were transfected with either pCMV-HA-c-Myc or pCMV-HA vector. At 48 h post-transfection, the cells were harvested, and c-Myc protein was detected by western blotting using an anti-HA tag antibody (top panel). β-actin was used as a loading control (bottom panel).
